# *Trichuris trichiura* egg extract proteome reveals potential diagnostic targets and immunomodulators

**DOI:** 10.1371/journal.pntd.0009221

**Published:** 2021-03-24

**Authors:** Katalina Cruz, Antonio Marcilla, Patrick Kelly, Michel Vandenplas, Antonio Osuna, María Trelis

**Affiliations:** 1 One Health Center for Zoonoses and Tropical Veterinary Medicine, Ross University School of Veterinary Medicine, Basseterre, St Kitts and Nevis; 2 Institute of Biotechnology, Biochemistry and Molecular Parasitology Group; Department of Parasitology, University of Granada, Granada, Spain; 3 Area of Parasitology, Department of Pharmacy and Pharmaceutical Technology and Parasitology, Universitat de València, Valencia, Spain; 4 Joint Research Unit on Endocrinology, Nutrition and Clinical Dietetics, Universitat de València—Health Research Institute La Fe, Valencia, Spain; PUCRS, BRAZIL

## Abstract

Embryonated eggs are the infectious developmental stage of *Trichuris trichiura* and are the primary stimulus for the immune system of the definitive host. The intestinal-dwelling *T*. *trichiura* affects an estimated 465 million people worldwide with an estimated global burden of disease of 640 000 DALYs (Disability Adjusted Life Years). In Latin America and the Caribbean, *trichuriasis* is the most prevalent soil transmitted helminthiasis in the region (12.3%; 95% CI). The adverse health consequences impair childhood school performance and reduce school attendance resulting in lower future wage-earning capacity. The accumulation of the long-term effects translates into poverty promoting sequelae and a cycle of impoverishment. Each infective *T*. *trichiura* egg carries the antigens needed to face the immune system with a wide variety of proteins present in the shell, larvae’s surface, and the accompanying fluid that contains their excretions/secretions. We used a proteomic approach with tandem mass spectrometry to investigate the proteome of soluble non-embryonated egg extracts of *T*. *trichiura* obtained from naturally infected African green monkeys (*Chlorocebus sabaeus*). A total of 231 proteins were identified, 168 of them with known molecular functions. The proteome revealed common proteins families which are known to play roles in energy and metabolism; the cytoskeleton, muscle and motility; proteolysis; signaling; the stress response and detoxification; transcription and translation; and lipid binding and transport. In addition to the study of the *T*. *trichiura* non-embryonated egg proteome, the antigenic profile of the *T*. *trichiura* non-embryonated egg and female soluble proteins against serum antibodies from *C*. *sabaeus* naturally infected with trichuriasis was investigated. We used an immunoproteomic approach by Western blot and tandem mass spectrometry from the corresponding SDS-PAGE gels. Vitellogenin N and VWD and DUF1943 domain containing protein, poly-cysteine and histidine tailed protein isoform 2, heat shock protein 70, glyceraldehyde-3-phosphate dehydrogenase, actin, and enolase, were among the potential immunoactive proteins. To our knowledge, this is the first study on the *T*. *trichiura* non-embryonated egg proteome as a novel source of information on potential targets for immunodiagnostics and immunomodulators from a neglected tropical disease. This initial list of *T*. *trichiura* non-embryonated egg proteins (proteome and antigenic profile) can be used in future research on the immunobiology and pathogenesis of human trichuriasis and the treatment of human intestinal immune-related diseases.

## Author summary

Who came first the worm or its egg? In the case of whipworm, we know it is the egg. The infective life cycle stage of the human whipworm (*Trichuris trichiura*) is the primary stimulus for the immune system of the definitive host. Each infective whipworm egg carries the information needed to face the immune system of the host with a wide variety of proteins present in the shell, larvae’s surface, and the accompanying fluid that contains their excretions/secretions. We investigated the soluble proteins of the non-embryonated egg using an immunoproteomic approach and then selected the top five proteins using a series of bioinformatic analysis. We used these top five proteins to recognize potential targets for immunodiagnostics and immunomodulation while comparing them to known female worm proteins. We found that the proteins we selected were involved in lipid transport, energy and metabolism, and muscle and motility. One protein has unknown function.

## Introduction

*Trichuris trichiura* is one of the major soil-transmitted helminths, along with roundworms (*Ascaris lumbricoides*) and hookworms (*Necator americanus* and *Ancylostoma duodenale*). It affects 465 million people worldwide with an estimated global burden of disease of 640,000 DALYs (Disability Adjusted Life Years) [1] and 337,000 YLDs (Years Lost to Disability) [2]. Following the accidental ingestion of the embryonated egg, larvae hatch in the proximal small bowel and migrate aborally to the colon and cecum, where they remain attached to the mucosa. They mature to adults in 30–120 days and can survive for 1 to 8 years [3,4]. After copulation, the females lay eggs 50–60 μm in length and 20–30 μm wide that are expelled in the feces in the non-infective form. They do not develop in direct sunlight and perish below 9°C but when exposed to appropriate environmental conditions of temperature and humidity [3,5] larvae develop over 20–30 days [3] and the eggs become the infectious life stage.

Trichuriasis in people is often asymptomatic, but it can manifest with abdominal pain, diarrhea, and in severe cases, a dysentery syndrome. Children are more commonly affected; heavy infections can result in rectal prolapse, severe anemia, stunted growth and poor school performance [5–8]. The severity of the symptoms not only depends on the parasite load but also on co-infections, immune-competence and past infections [4,9].

Diagnosis of infections is usually based on the detection of eggs through coprological analyses, but such techniques lack sensitivity and do not predict true parasite loads or real time infection status due to the dynamic events inherit in the life cycle of the nematode [9–11]. False negative results can occur during the prepatent period, in single sex infections, low level infections, and when females are not releasing eggs at the time of sample collection [4,9,12]. There is thus an important need for an alternative indirect diagnostic method with greater sensitivity.

While somatic and excretion/secretion products from adult *T*. *trichiura* have been studied in depth and shown to elicit protective immune responses which could be useful in immunodiagnostics [13–16], immunogens of other life-cycle stages of the parasite, such as the eggs, have not been thoroughly investigated. Eggs contain the first antigens of *T*. *trichiura* that are presented to a naïve host’s immune system and would thus seem to be the most important in the development of an early and effective immune response to limit infection. In the few reports that are available on egg antigens of other parasites, evidence has been presented that eggs may be sources of diagnostic antigens and modulators of the immune system [17,18]. For example, the *S*. *mansoni* egg secretome revealed the identification of proteins actively secreted by live schistosome eggs providing novel information to improve the understanding of immune modulation and the pathology of infections [17]. Interestingly, the administration of embryonated eggs from the animal species *Trichuris suis* or *Trichuris muris* as immunotherapy to humans have been described to downregulate aberrant intestinal inflammation and potentially be of use in immune-related intestinal conditions such as chronic intestinal inflammatory diseases [6,19–22]. Their immunomodulatory capacity continues to be investigated with some studies trying to identify the molecules responsible for those effects [14,22–25].

To characterize the proteins in non-embryonated (NE) eggs of *T*. *trichiura* which might be used for new immunodiagnostic techniques or in immunomodulation therapies we studied the soluble NE egg extract proteome using a stage-specific proteomic approach with SDS-PAGE, Western blot and Liquid Chromatography with Tandem Mass Spectrometry (LC-MS/MS). Detailed information about the specific proteins and functional analysis of the molecules within the trichuris NE egg provide the first insight into their intricate role within the life cycle and the interaction with the host. Our characterization of the NE egg-derived proteins complement work with *T*. *suis* [6,21] and *T*. *muris* [26,27] models that focus on the prevention of autoimmune diseases and the development of new immunodiagnostic techniques.

The African Green monkey (AGMs) as a naturally infected host accurately predicts the evaluation of parasitic diseases due to the similarities in antigen species to those affecting humans [28,29]. Being naturally infected with *T*. *trichiura*, the St. Kitts green monkey serves as a predictive disease modeling test system and facilitates preclinical evaluation having similar immunological responses as humans.

## Methods

### Ethics statements

Samples were collected from AGMs enrolled in other studies approved by the Institutional Animal Care and Use Committee of the Biomedical Research Foundation and Virscio on February 26, 2018 (approval number: AC18175).

### Sera samples

Whole blood samples were collected from 10 AGMs naturally infected with *T*. *trichiura* [28,29] as part of other studies and transferred to 5 mL vacutainers (Covidien Monoject, Massachusetts, USA). Sera were separated immediately by centrifugation at 2,000 g for 15 min at 4°C and stored at -80°C until thawed at room temperature and pooled for analysis as below.

### *T*. *trichiura* adults

Adult worms were obtained at necropsy from the large intestine of naturally infected animals humanely euthanized as part of other IACUC approved studies. The large intestine was placed in 0.9% saline solution for around 2 h at room temperature (30–32°C) to allow the *T*. *trichiura* to detach from the mucosa. Thereafter, the large intestine was opened and washed over a 100 μm sieve before trapped contents were examined under a stereomicroscope (7x – 10x magnification) for the presence of *T*. *trichiura* adults, which were isolated, sexed and preserved at—80°C.

### *T*. *trichiura* non-embryonated (NE) egg extract

Uteri were removed from 50 *T*. *trichiura* females using a 30G ½” needle (BD Microlance, Fraga, Huesca, Spain) (10x – 30x magnification) and placed in phosphate buffered saline (PBS; pH 7.4). The uteri were opened with a longitudinal incision to facilitate the release of non-embryonated eggs which were pooled. After five washes in PBS (10,000 g; 1 min) the supernatant was removed and a 1% protease inhibitors cocktail (Complete mini EDTA-free, Roche, Berlin, Germany) with 1% Triton X-100 (Sigma-Aldrich, Steinheim, Germany) in PBS added to the egg pellet which was homogenized as described previously [30]. To ensure disruption of *Trichuris* eggshells, the homogenate was sonicated while frozen at -20°C using ten cycles of 10x 1-second pulses at maximum intensity with a Microson Ultrasonic Cell Disruptor XL (Misonix, Farmingdale, NY, USA). Homogenates were checked for egg disruption under a stereomicroscope, centrifuged (10,000 g; 10 min at 4°C), and the supernatant containing the soluble NE egg proteins recovered (the *T*. *trichiura* NE egg extract—EE). The EE total protein concentration was determined by a commercial Protein Assay (Bio-Rad, Hercules, USA) based on the Bradford method of quantification of soluble proteins [31] and stored frozen at -20°C until further analysis.

### *T*. *trichiura* female extract (FE)

As part of the comparative study, the *T*. *trichiura* female extract (FE) was analyzed in parallel. Fifty female adults were obtained from the intestines of naturally infected AGMs, washed five times in PBS and homogenized with a Teflon homogenizer in PBS containing a 1% protease inhibitors cocktail (Complete mini EDTA-free, Roche) with 1% Triton X-100 (Sigma-Aldrich, Steinheim, Germany) in PBS. After initial centrifugation at a low speed to remove larger particles, the homogenate was centrifuged again (15,000 g; 30 min at 4°C), and the supernatant collected and stored frozen at -20°C until further analysis. The protein content was measured in the same way as the EE.

### One dimensional SDS-PAGE

*T*. *trichiura* EE (10 μg/well) and FE (10 μg/well) were diluted in Laemmli buffer (4X) (Bio-Rad) (1:1), denatured at 100°C for 5 min and separated by one dimensional gel electrophoresis (1-DE) in Mini-Protean TGX precast acrylamide gels (4–15% gradient, 10 well comb, 50 μL/well) (Bio-Rad) under reducing conditions with 80–120 V in a Mini-PROTEAN Tetra System electrophoresis system (Bio-Rad) as previously described [32]. Samples were run simultaneously with molecular weight markers (4 μL) (Precision Plus Protein Dual Color Standards, Bio-Rad).

The gels were stained with Coomassie brilliant blue to analyze the protein patterns and the most prominent bands excised for proteomic analysis. Before staining, the gels were fixed (50% methanol and 10% glacial acetic acid) overnight with gentle agitation (solution changed once after 1 h). Gels were stained (0.1% Coomassie brilliant blue R-250, 50% methanol and, 10% glacial acetic acid) for 20 min with gentle shaking before destaining (40% methanol and 10% glacial acetic acid) with repeated changes of the solution until the gel background was clear. Gels were stored at 4°C in 5% glacial acetic acid.

### Western blot

For immunoblotting, following one dimensional electrophoresis, proteins were transferred onto nitrocellulose paper using a Trans-Blot Turbo transfer system (Bio-Rad) for 7 min. The blotted membrane was blocked with 5% skimmed milk in 0.05% PBS-Tween 20 (PBST) for 2 h at room temperature and, after successive washes in PBST, incubated overnight at 4°C with a pool of AGMs serum samples diluted 1:500 in PBST. After three washes for 30 min in PBST, the membranes were incubated for 4 h at room temperature with the secondary antibody (peroxidase-labeled goat anti-primate IgG (Novusbio, Colorado, USA) (1: 5,000 in PBST). Finally, membranes were washed three times in PBST for 30 min each and the assay developed using Clarity Western ECL substrate (Bio-Rad) mixed in a 1:1 ratio. The positive reactions were determined by the appearance of clearly defined protein bands detected by chemiluminescence with an Amersham Imager 600 (GE Healthcare, New Jersey, USA). The relative molecular masses of the recognized protein fractions were determined by comparison with molecular weight markers (kDa), and data analysis was completed as previously described [33].

### Proteomic analysis of the *T*. *trichiura* egg extract (EE)

#### Sample preparation

Following electrophoresis and staining, a complete gel strip of EE was cut and digested with 500 ng of sequencing grade trypsin (Promega, Wisconsin, USA) in 200 μL of ammonium bicarbonate solution as described elsewhere [34]. The selected EE and FE bands from other gels (egg and female extracts) were manually excised and digested with 100 ng of sequencing grade trypsin (Promega) in 100 μL of ammonium bicarbonate as described elsewhere [34]. Digestion was stopped with 1% trifluoracetic acid (TFA), and a double extraction with acetonitrile (ACN) was performed. The final peptide solutions were vacuum-dried and resuspended with 25 μL of 2% ACN and 0.1% TFA (pH 2.0) for the EE and 9 μL of 2% ACN and 0.1% TFA (pH 2.0) for the individual EE and FE bands as previously described [32].

#### Liquid chromatography and tandem mass spectrometry (LC-MS/MS)

Liquid chromatography and tandem mass spectrometry were performed at the Proteomics facility of Servei Central de Suport a la Investigació Experimental (SCSIE) of Universitat de València (Burjassot, Spain).

To initiate the elution process, 5 μL of the final peptide solution was loaded onto a trap column (Nano-LC Column, 3 μm C18-CL, 350 μm x 0.5 mm, Eksigen, AB Sciex, California, USA) and desalted with 0.1% TFA at 3 μL / min for 5 min. The peptides were loaded onto an analytical column (LC Column, 3 μm C18-CL, 75 μm x 12 cm, Nikkyo, Nikkyo Technos Co., Ltd. Tokyo, Japan) equilibrated in 5% acetonitrile, 0.1% formic acid (FA) and eluted using a linear gradient (5–35%) of solvent B (0.1% FA in ACN) in A (0.1% FA) for 120 min for the EE and 30 min for the individual FE bands at a flow rate of 300 nL/min. The eluted peptides were analyzed with a nanoESI-Q-TOF mass spectrometer (5600 TripleTOF, AB Sciex) in an information dependent acquisition mode (IDA). The eluted sample was ionized applying 2.8 kV to the spray emitter, and survey MS1 scans were acquired from 350 to 1250 m/z for 250 ms. The quadruple resolution was set to ‘UNIT’ for MS2 experiments, which were acquired from 100 to 1,500 m/z for 50 ms in ‘high sensitivity’ mode. The following switch criterion was used: charge 2+ to 5+, minimum intensity, 70 counts per second (cps). Up to 50 ions were selected for fragmentation after each survey scan. Dynamic exclusion was set to 15 s. The system sensitivity was controlled with 2 fmol of 6 proteins (LC Packings, A Dionex Company, Amsterdam, Netherlands).

### Bioinformatics

ProteinPilot Software 4.5.1 revision 2768 (AB Sciex) utilizing the Paragon algorithm 4.5.1.0 revision 2765 (AB Sciex) with default parameters was used to generate a peak list directly from 5600 TripleTof.wiff files. All.wiff files from the samples were combined in a single search. The Paragon Algorithm included in ProteinPilot software was used for searching the NCBI protein database (version 01–2016) with the following parameters: tryptic specificity, cys-alkylation, Metazoa, Nematoda, and *T*. *trichiura* protein taxonomy restrictions. These are typical parameters used for baseline proteomic analysis adjusted to the specific species of interest.

Protein grouping was done by Pro Group algorithm (a set of proteins that share physical evidence guided by observed peptides only) and identification was considered accurate when the ProteinPilot unused score was > 1.3 corresponding to a 95% confidence according to the following equation: ProtScore = -log (1-(percent confidence/100)).

Protein identification was conducted against the *T*. *trichiura* adult proteome on the Parasite WormBase (version of 2017–262 05—WormBase - www.parasite.wormbase.org). All identified proteins were subsequently assigned to the UniProt database and classified in Gene Ontology (GO) (https://www.uniprot.org) according to their molecular function and biological processes.

## Results

Our results are the first report of the proteome of soluble egg extracts of *T*. *trichiura* from AGMs (*C*. *sabaeus*) and describe the potential immunomodulators and antigens recognized by sera of naturally infected animals.

### Proteomic characterization of the *T*. *trichiura* egg extract (EE)

With the spectrometric data obtained using ProteinPilot software v4.5 we identified 246 proteins. The unique peptide sequence transcript identification code obtained from the spectrometric data and their respective accession number from WormBase (https://parasite.wormbase.org) enabled us to characterize 231 of the 246 proteins found (S1 Table): 212 had significant homologies with known *T*. *trichiura* adult stage proteins and 19 were novel uncharacterized proteins with unknown ontology. The remaining 15 proteins generated a unique peptide sequence transcript identification code but yielded no accession number from https://parasite.wormbase.org and were thus excluded from further study as no further information could be obtained based on the current genome available.

### Gene ontology (GO)

**Fig 1:**
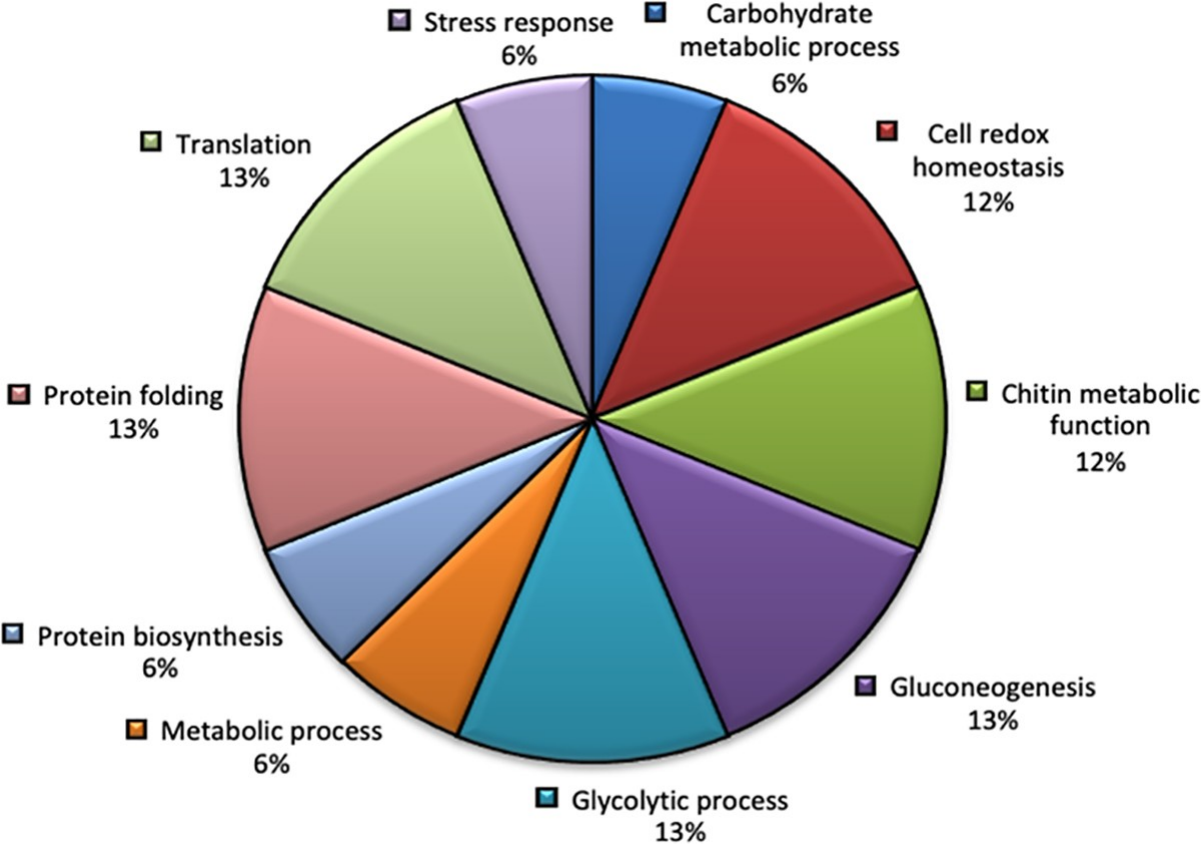
Main biological processes of the identified proteins in the non-embryonated egg extract proteome (EE) of *T*. *trichiura* according to information obtained from the Gene Ontology (GO) database https://www.uniprot.org.

When the proteins were categorized according to their molecular function described in the Gene Ontology (GO) database (UNIPROT; https://www.uniprot.org), 168 were found to have known functions (S1 Table). The different functional groups and biological processes of the most representative proteins of our analysis (with 10 or more distinct peptides) are shown in Table 1 and Fig 1. Only a single annotation was assigned to a given protein. Functional annotation of the identified proteins was assigned using GO, which revealed functionally diverse molecules of the common protein families or groups: energy and metabolism; cytoskeleton, motility and muscle; proteolysis; signaling; stress and detoxification; transcription and translation; and lipid binding and transport (Table 1). Their specific molecular functions range from molecules involved in ATP, actin, carbohydrate, chitin, lipid and magnesium ion binding, as well as molecules that take part in oxidoreductase, aminopeptidase, glycogen phosphorylase and metallopeptidase activity (Table 1). Others include lipid transporter, motor and protein disulfide isomerase activity, together with structural constituents of the ribosome or proteins associated with the elongation phase of protein synthesis. Proteins with kinase and intracellular cholesterol transport functions were also identified (Table 1). The most abundant category for the biological process assigned to the egg proteins were protein folding, translation, gluconeogenesis, and glycolytic process all equally represented (13%), followed by cell redox homeostasis (12%), chitin metabolic function (12%), and to a lesser extent metabolic processes, carbohydrate metabolic processes, protein biosynthesis and stress responses (Fig 1).

**Table 1:** Main proteins identified in the EE (10 or more distinct peptides) organized by functional annotation. Only a single annotation was assigned to a given protein.

Functional annotation	Molecular function*	Acc. No. Wormbase	% Cov.	Peptides (95%)	MW (KDa)	Signal peptide	Biological process*
**Energy and metabolism**							
Alpha-1,4 glucan phosphorylase	Glycogen phosphorylase activity	A0A077YWK8	20.29	14	101.447	-	carbohydrate metabolic process
ECH domain containing protein	Catalytic activity	A0A077Z1N9	44.83	17	31.202	-	metabolic process
Enolase	Magnesium ion binding	A0A077YX57	44.49	27	49.513	-	glycolytic process
Glyceraldehyde-3-phosphate dehydrogenase	Oxidoreductase	A0A077ZHV3	56.10	66	37.536	-	glycolytic process
Malic enzyme	Oxidoreductase	A0A077Z5U2	28.04	13	62.847	-	Unknown
Phosphoenolpyruvate carboxykinase GTP	Kinase	A0A077Z7M0	29.04	20	70.975	-	gluconeogenesis
Triosephosphate isomerase	Isomerase	A0A077ZC84	57.26	10	27.399	-	gluconeogenesis
**Cytoskeleton, motility and muscle**							
Actin	ATP binding	A0A077ZE37	55.59	35	41.838	-	unknown
Actin 5C	ATP binding	A0A077YWW9	53.66	29	41.036	-	unknown
Epididymal secretory protein E1	Intracellular cholesterol transport	A0A077Z0I4	43.44	28	45.783	1 to 23	unknown
Intermediate filament protein IFA 1	Unknown function	A0A077Z6U0	23.39	14	70.711	-	unknown
Moesin-ezrin-radixin 1	Actin binding	A0A077ZIT0	25.97	12	55.989	-	unknown
Paramyosin	Motor activity	A0A077Z8E1	38.61	30	101.488	-	unknown
Tropomyosin	Unknown function	A0A077ZIM1	41.20	38	87.298	-	unknown
**Proteolysis**							
Cytosol aminopeptidase	Aminopeptidase activity	A0A077Z3I7	23.80	10	54.409	-	unknown
Peptidase M13 and Peptidase M13 N domain containing protein	Metalloendopeptidase activity	A0A077ZJE5	24.05	14	81.361	-	unknown
**Signaling**							
78 kDa glucose regulated protein	ATP binding	A0A077Z8G8	22.58	12	72.784	1 to 18	unknown
CBM 14 domain containing protein	Chitin binding	A0A077Z111	46.72	38	95.908	-	chitin metabolic function
CBM 14 domain containing protein	Chitin binding	A0A077Z8B3	28.37	18	78.597	-	chitin metabolic process
Galectin	Carbohydrate binding	A0A077YZM7	50.72	27	31.967	-	unknown
Galectin	Carbohydrate binding	A0A077ZG03	39.64	25	32.25	-	unknown
**Stress and detoxification**							
Chaperonin protein heat shock protein 60	ATP binding	A0A077ZIE8	28.00	11	62.806	-	protein folding
Heat shock protein 70	L-malate dehydrogenase activity	A0A077Z8E4	20.07	21	130.299	-	stress response
Heat shock protein 90	ATP binding	A0A077Z1F6	17.08	12	82.924	-	protein folding
Protein disulfide-isomerase	Protein disulfide isomerase activity	A0A077ZJZ3	35.03	14	55.125	1 to 18	cell redox homeostasis
Protein disulfide-isomerase	Protein disulfide isomerase activity	A0A077ZLF1	35.95	15	55.73	1 to 16	cell redox homeostasis
Superoxide dismutase [Cu-Zn]	Oxidoreductase	A0A077Z345	69.86	12	15.274	-	unknown
**Transcription and Translation**							
40S ribosomal protein SA	Structural constituent of ribosome	A0A077YZD4	42.57	13	34.141	-	ribosomal small subunit assembly, translation
Elongation factor 1-alpha	Elongation factor	A0A077YYL7	33.48	12	51.086	-	protein biosynthesis
Mediator of RNA polymerase II transcription subunit 22	Protein disulfide isomerase activity	A0A077Z2H0	69.06	17	15.485	1 to 19	cell redox homeostasis
Ribosomal L18p and L18 c domain containing protein	Structural constituent of ribosome	A0A077ZPB6	42.67	13	35.744	-	translation
**Lipid binding and transport**							
Vitellogenin N and VWD and DUF1943 domain containing protein	Lipid transporter activity	A0A077ZE83	56.35	205	198.527	1 to 19	unknown
Uncharacterized protein	Lipid binding	A0A077ZMT5	14.14	20	84.314	-	unknown
**Others**							
DUF290 domain containing protein	Unknown function	A0A077Z8H2	43.67	20	17.876	1 to 19	Unknown
Poly-cysteine and histidine tailed protein isoform 2	Unknown function	A0A077Z5Q5	50.79	109	50.494	-	Unknown
Protein asteroid	Unknown function	A0A077Z2C7	63.64	34	30.674	1 to 23	Unknown
Transthyretin-like protein 46	Unknown function	A0A077Z9N4	42.57	12	16.458	1 to 18	Unknown
Uncharacterized protein	Unknown function	A0A077YXT2	16.08	10	69.581	1 to 18	unknown
Uncharacterized protein	Unknown function	A0A077YX18	20.42	10	32.73	1 to 18	unknown
Uncharacterized protein	Unknown function	A0A077Z544	48.83	19	33.553	1 to 23	unknown

* Molecular function and biological process was obtained from the Gene Ontology (GO) database.

### 1-DE and immunoblot analysis of *T*. *trichiura* EE and FE

To identify the species-specific parasite antigens, 1-DE SDS-PAGE and Western blots were performed on EE and FE with serum from naturally infected AGMs.

**Fig 2:**
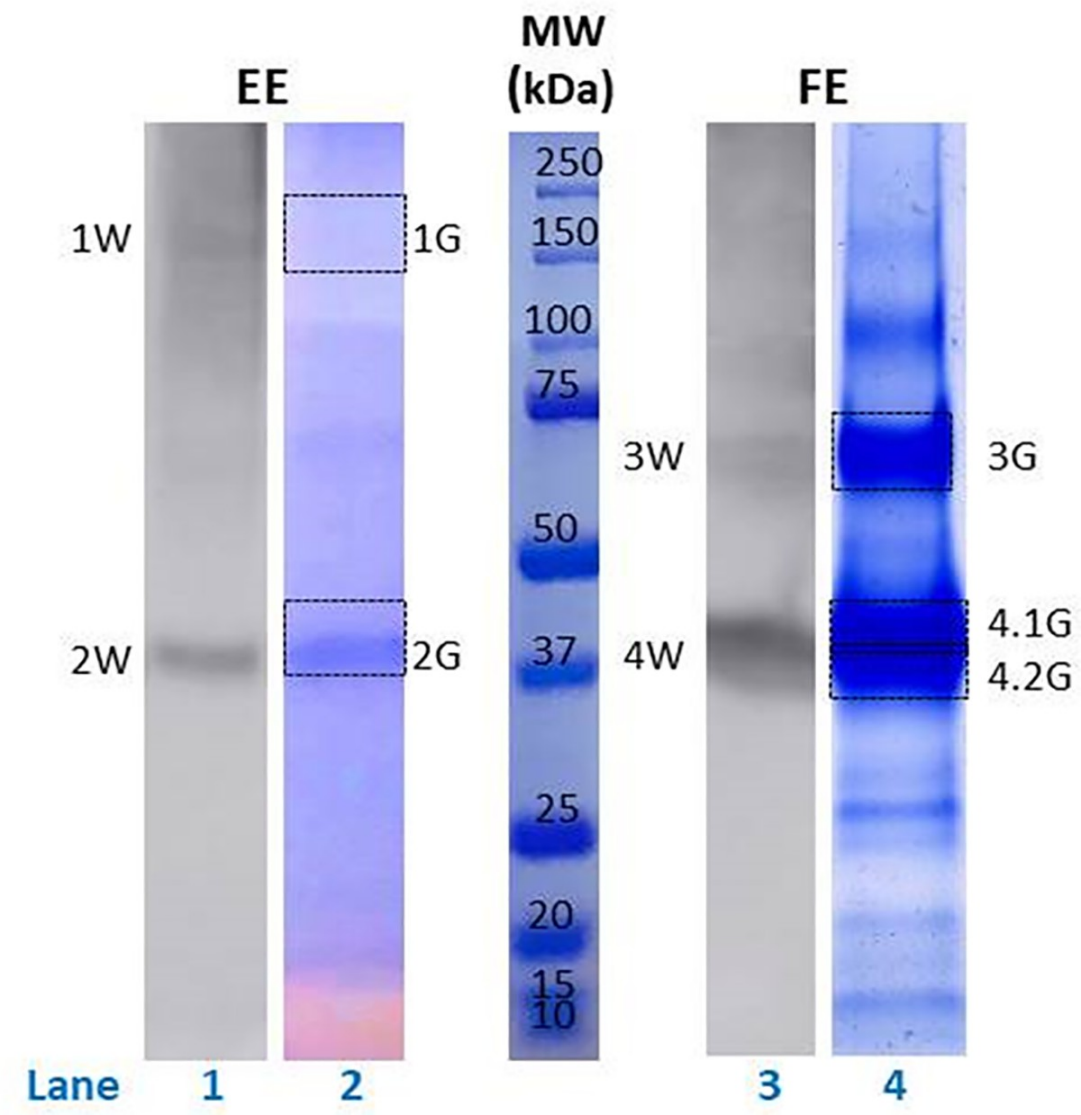
Major immunogenic proteins detected in *T*. *trichiura* extracts. Western blot showing AGMs sera antibodies response to *T*. *trichiura* egg extract (EE) (Lane 1) and female extract (FE) (Lane 3) (10 μg/lane). Bands 1W-2W and 3W-4W indicate the regions containing antigens recognized most strongly by sera antibodies in EE and FE, respectively. Corresponding SDS-PAGE of EE (Lane 2) and FE (Lane 4), stained with Coomassie Brilliant Blue R-250 and excised areas of each, 1G-2G and 3G-4.1G-4.2G, containing the most immunogenic peptides for proteomic analysis. Molecular weight in kDa is lane labeled as MW.

For the EE, the possible identity of the antigens revealed in Western blots (Fig 2, Lane 1) was investigated by matching the molecular weights of the bands seen with those of proteins identified in the EE proteome and are presented in Table 2. In addition, the same specific areas on SDS-PAGE gels (1G and 2G) (Fig 2, Lane 2) corresponding to the bands in the Western blots were excised and used for confirmatory proteomic analysis by LC-MS/MS and presented in Table 3.

**Table 2:** Potential identity of the EE proteins targeted by serum antibodies based on the MW data of the EE proteome.

Accession number	Annotation	MW (kDa)	Peptides* (95%)
**Band 1W (≈ 170 kDa)**			
A0A077ZE83	Vitellogenin N and VWD and DUF1943 domain containing protein	198.527	205
A0A077Z8E4	Heat shock protein 70	130.299	21
**Band 2W (≈ 37 kDa)**			
A0A077Z5Q5	Poly-cysteine and histidine tailed protein isoform 2	50.494	109
A0A077ZHV3	Glyceraldehyde-3-phosphate dehydrogenase	37.536	66
A0A077ZE37	Actin	41.838	35
A0A077YWW9	Actin 5C	41.036	29
A0A077YX57	Enolase	49.513	27
A0A077Z0I4	Epididymal secretory protein E1	45.783	28

*The number of distinct peptides having at least 95% confidence.

**Table 3:** Protein identities, in decreasing abundance, in immunodominant bands 1W and 2W in Western blots with EE as antigen. Proteins were identified by LC-MS/MS of corresponding areas in SDS-PAGE gels, 1G and 2G.

Accession number	Annotation	MW (kDa)	Peptides* (95%)
**Area 1G (≈ 150–200 kDa)**			
A0A077ZE83	Vitellogenin N and VWD and DUF1943 domain containing protein	198.527	241
A0A077Z8E4	Heat shock protein 70	130.299	3
**Area 2G (≈ 37–45 kDa)**			
A0A077Z5Q5	Poly-cysteine and histidine tailed protein isoform 2	50.940	66
A0A077YX57	Enolase	49.513	18
A0A077ZHV3	Glyceraldehyde-3-phosphate dehydrogenase	37.536	15
A0A077ZE37	Actin	41.838	14
A0A077Z3K7	Phosphoglycerate kinase	44.724	12
A0A077Z8X2	Dolichyl- diphosphooligosaccharide-protein glycosyltransferase 48 kDa subunit	48.787	12
A0A077ZF21	Adenosylhomocysteinase	47.827	10
A0A077Z3H9	Tubulointerstitial nephritis antigen	50.458	9
A0A077YXR0	Calponin domain containing protein	40.694	7
A0A077ZJA3	3 ketoacyl coenzyme A thiolase	43.402	6
A0A077YYL7	Elongation factor 1-alpha	51.086	6
A0A077Z1Z4	Serpin domain containing protein	43.328	6

*The number of distinct peptides having at least 95% confidence.

The immune-complexes identified by Western blot with the EE as antigen ranged from 37 to 200 kDa with the most immunogenic in two distinct bands, band 1W (≈ 170 kDa) and band 2W (≈ 37 kDa) (Fig 2, Lane 1).

For the FE, the comparative study for the specific immune-complexes identified by Western blot (Fig 2, Lane 3) and areas matched on SDS-PAGE gels (Fig 2, Lane 4) were also excised and analyzed by LC-MS/MS and presented in Table 4. With the FE as antigen these ranged from 33 to 70 kDa with the most immunogenic in two distinct bands, band 3W (≈ 60–70 kDa) (Fig 2, Lane 4) and band 4W (≈ 37 kDa) (Fig 2, Lane 3).

**Table 4:** Protein identities, in decreasing abundance within FE excised gel areas (3G, 4.1G, 4.2G) with suitable MW matching Western blot band areas 3W and 4W.

Accession number	Annotation	MW (kDa)	Peptides (95%)
**Area 3G (≈ 60–70 kDa)**			
A0A077ZIM1	Tropomyosin	87.298	19
A0A077ZIM7	Papilin	80.804	6
A0A077ZEY0	Calsequestrin	49.211	5
A0A077YX57	Enolase	40.513	5
**Area 4.1G (≈ 37–45 kDa)**			
A0A077Z5Q5	Poly-cysteine and histidine tailed protein isoform 2	50.494	65
A0A077ZE37	Actin	41.838	7
A0A077ZHV3	Glyceraldehyde-3-phosphate dehydrogenase	37.536	5
A0A077ZEY0	Calsequestrin	49.211	5
**Area 4.2G (≈ 33–37 kDa)**			
A0A077Z0I4	Epididymal secretory protein E1	45.783	8
A0A077Z0N1	Actin-depolymerizing factor 2, isoform c	35.411	4
A0A077Z5Q5	Poly-cysteine and histidine tailed protein isoform 2	50.494	4

Based on corresponding molecular weights in the EE proteome, band 1W contained vitellogenin N and VWD and DUF1943 domain containing protein (VgNVD) with 205 distinct peptides and heat shock protein 70 (HSP-70) with 21 distinct peptides (Table 2). Confirmatory LC-MS/MS of the corresponding Coomassie-stained band confirmed that VgNVD, with 241 distinct peptides, was the most representative protein within the 1W area (Table 3).

Analysis of band 2W identified poly-cysteine and histidine-tailed protein isoform 2 (PCHTP-2), glyceraldehyde-3-phosphate dehydrogenase (GAPDH), actin, actin 5C, enolase, and epididymal secretory protein E1 (Table 2) and confirmatory analysis with LC-MS/MS also indicating there were other proteins present such as phosphoglycerate kinase, the dolichyl-diphosphooligosaccharide-protein glycosyltransferase 48 kDa subunit, adenosylhomocysteinase, tubulointerstitial nephritis antigen, calponin domain containing protein, 3 ketoacyl coenzyme A thiolase, elongation factor 1-alpha and serpin domain containing protein (Table 3) while actin 5C and epididymal secretory protein E1 were not confirmed.

Regarding the comparative proteomic analysis of reactive areas displayed in FE Western blot, the analysis of the band 3G (≈ 60–70 kDa) (Fig 2, Lane 4), which corresponded to band 3W, revealed again PCHTP-2 as one of the proteins identified with the highest number of matching peptides. This protein was also identified in bands, 4.1G and 4.2G (Table 4). The proteomic results of both sections of the band 4W showed some proteins shared between the EE and FE, such as PCHTP-2, Actin and GAPDH, suggesting they are likely to be the major ones in both samples. This finding is not surprising, since most of the EE antigens are also present in FE (eggs contained in the uterus).

In comparing the Western blots with EE and FE as antigen, highly reactive bands were seen in both with a molecular weight of around 37 kDa (2W and 4W) (Fig 2, Lanes 1 and 3). There were intensely staining protein bands in the corresponding SDS-PAGE gels. The other two prominent bands seen in the Western blots of the EE and FE antigens, 1W and 3W, were of different molecular weights and thus seemed to be stage-specific to the different life stages (Fig 2). Antigenic band 1W was barely detected in the corresponding SDS-PAGE indicating a low concentration of protein, while band 3W corresponded to a prominent band on the corresponding SDS-PAGE gel indicating a high concentration (1G) (Fig 2, Lane 2). Although the 2W and 4W major antigenic bands of the EE and FE, respectively, both had a molecular weight of 37 kDa and shared several proteins (PCHTP-2, Actin and GAPDH), there were also specific proteins which appeared in only the egg or adult female stage. Dolichyl-diphosphooligosaccharide-protein glycosyltransferase 48kDa subunit, adenosylhomocysteinase, tubulointerstitial nephritis antigen, calponin domain containing protein, 3 ketoacyl coenzyme A thiolase, elongation factor 1-alpha and serpin domain containing protein appeared as typical of EE (Table 3). Meanwhile, Calsequestrin, Epididymal secretory protein E1, and Actin-depolymerizing factor 2-isoform c were only present in FE (Table 4).

## Discussion

### *T*. *trichiura* non-embryonated egg proteome

#### Diagnostic challenges to overcome

Recent reports confirm that most cases of *T*. *trichiura* infections remain undiagnosed [9], and chronic infections can remain undetected for years [4]. This is because diagnosis of infections is based on the detection of eggs through coprological analyses that do not predict true parasite loads or real-time infection status. Early diagnosis of trichuriasis and diagnostic methods that do not rely on inconsistent clinical signs or fecal analysis are crucial to detect the infections following accidental ingestion and during the prolonged prepatent period of the parasite. At present, there are only limited data on *T*. *trichiura* antigens that can be used in serological diagnostic tests and the purpose of this study is to present the first description of the *T*. *trichiura* non-embryonated egg proteome and the immunodominant proteins present in both EE and FE. We also consider that the method described for *T*. *trichiura* egg isolation would be suitable for isolating large amounts of eggs from a more sterile and practical environment than the feces and, although the in-uteri eggs are not yet embryonated, they do present somatic and excretory/secretory proteins of the egg shell and those of dividing embryonic cells. We are demonstrating that their immunoproteomic analysis provides valuable information that warrants further study.

#### *T*. *trichiura* genome and other helminths provide insights into the egg proteome

In *T*. *trichiura*, the parasite-host interactions are poorly understood and are highly influenced by the parasite’s life cycle. Limited information on stage-specific antigens, immune evasion strategies and immunomodulatory effects have been described in animal models of *T*. *muris* and *T*. *suis* [21,26,27]. Foth and collaborators [15] described the whole-genome of the human-infective adult *T*. *trichiura* and we can now compare the *T*. *trichiura* egg proteome to their findings; they also identified numerous genes that are differentially expressed in a sex- or stage-specific manner. The most abundant transcripts found in this extensive study included proteins we have now definitively identified in the EE proteome, such as two WAP domain containing SLP-like proteins, protease inhibitors such as cystatin-domain containing protein and nematode cuticle collagen N-terminal domain containing proteins and chitin binding domain containing proteins such as CBM14 domain containing proteins (Tables 1 and S1).

Furthermore, with more or less representation, but of particular interest within the context of the present work, we have found *Trichuris* egg proteins with known immunomodulatory properties such as macrophage migration inhibitory factor homolog (MIF) (S1 Table), previously identified in *T*. *trichiura* adult [14], and 14-3-3 protein (S1 Table) which has also been identified in several developmental stages of other nematodes, *Trichinella britovi* [35] and *Trichinella spiralis* [36] and trematodes, *Schistosoma japonicum* [37]. Both proteins are considered as enhancers of humoral and cellular immune responses [38]. Although their function and biological process in *T*. *trichiura* remains unknown we are confirming the presence in the EE proteome and highlighting the potential role in the initial stages of the parasite-host interaction.

#### EE proteome proteins with the largest numbers of distinct peptides

*Lipid transporter and major secreted protein with unknown function*. Interestingly, two of the proteins identified with the largest numbers of distinct peptides in the EE proteome presented in this study, VgNVD and PCHTP-2 (Table 1), were also among the top 25 most abundant transcripts found by Foth and collaborators [15]. Vitellogenins are a lipid transfer proteins, they play a significant role in embryonic development and are extensively conserved amongst nematodes [39]. They provide the growing embryo with amino acids [40], therefore VgNVD being the most abundant protein in the EE proteome represents an important antigenic target that can be consistently identified in eggs and adult females. The detection of PCHTP-2 in the EE proteome as the second most frequently detected protein is in accordance with Shears and collaborators [26] who found it to be the most abundant protein in the *T*. *muris* adult secretome. Even though a specific function has not been assigned yet for *T*. *trichiura*, Bancroft and collaborators [27] identified PCHTP-2 as the most abundant protein in cecal mucus from chronically infected mice with *T*. *muris* and confirmed its expression in all developmental stages confirming PCHTP-2 as the major secreted protein of the whipworm despite not presenting signal peptide.

*Energy and metabolism*. One of the most represented groups of proteins we found in the egg proteome were those related to energy and metabolism and included proteins associated with glycolysis (enolase and glyceraldehyde-3-phosphate dehydrogenase (GADPH), gluconeogenesis (triosephosphate isomerase and phosphoenolpyruvate carboxykinase GTP) and other metabolic enzymes such as alpha-1,4 glucan phosphorylase and malic enzyme (S1 Table). This fact is consistent with previous studies in which these metabolic enzymes were described on the surface of the helminths, nematodes, and trematodes, found to participate in oxidative processes, parasite invasion and migration processes within the host [32,33,41–44].

*Muscle*, *motility and cytoskeleton*. The ensuing functional group with the largest number of representatives was related to the cytoskeleton, muscle and motility. Actin, tropomyosin, paramyosin, intermediate filament protein IFA 1 and epididymal secretory protein E1 were found with a high number of distinct peptides (S1 Table). These proteins are essential to enhance the motility of nematodes and have also been recorded in many helminthic proteomes: somatic extract of adults of *T*. *spiralis* [45], *T*. *britovi* [35], *Syphacia muris* [44], and *Echinostoma caproni* [46]; and in egg secretions of *Schistosoma mansoni* [17]. Specifically, intermediate filament protein IFA1 has been studied in *Caenorhabditis elegans*, demonstrating that in nematodes and potentially similar for *T*. *trichiura*, they allow epidermal elongation in the larval stages to grow into adults [47].

*Survival*: *antioxidants and chaperones*. We also found proteins essential for the survival of the nematode within its host, in the hostile conditions of the cecum, during stress and for detoxifying processes including antioxidants and chaperones. The Cu/Zn superoxide dismutase (Cu/Zn-SOD) was found in the EE proteome (S1 Table) and has also been identified on the adult surface and larval extracts (secreted and somatic) of *Toxocara canis* [38], in the somatic extract of adults of *Fasciola hepatica*, and the *S*. *mansoni* egg secretome [17,48]. This essential enzyme antagonizes the host’s inflammatory responses by regulating the free radical balance and reactive oxygen species in cells protecting helminths against cell death [49]. Heat shock proteins (HSP90, HSP70, HSP60) are inducible conserved proteins widely described in parasite proteomes and secretomes, and we have confirmed their presence in the EE proteome. They act as molecular chaperones which fold, assemble and translocate other proteins to ensure the survival of the parasite by defending it against stressful situations being important in stress tolerance [50]. Small heat shock proteins HSP20 and HSP20 domain containing protein were also identified in EE proteome (S1 Table), which are known to aid parasite survival under hostile conditions such as heat or nutritional stress [51].

*Signaling*. Within the proteins implicated in signaling pathways, we identified galectin in the EE proteome, a type of lectin found in different extracts of nematodes such as adults and larvae of *T*. *canis* [38] and extract of infective larvae (L3) of *Haemonchus contortus* [52] with a role in immune signaling pathways. Nematode galectins are believed to be immunological mediators with implications in survival and interaction with the host [53] and modulate a range of immune responses, including the cellular immune response, inflammatory processes and immune regulation [54] all essential for prolonged survival of *T*. *trichiura* in the host.

### Antigenic profile of *T*. *trichiura* EE and FE extracts and identification of the top 5 immunodominant proteins

Previous studies have used an immunoproteomic approach to determine the antigenic proteins of helminths at different developmental stages (larvae and adults) and the serological responses to soluble protein extracts of *Ascaris lumbricoides* [55], *T*. *britovi* [35], *Schistosoma japonicum* [56] and *Taenia solium* [57].

Parasitic worms, like *T*. *trichiura* have a remarkable ability to modulate the host immune response through several mechanisms; specific parasite-derived proteins can modulate immune functions playing an essential role in the parasite-host interaction. Excretion/secretion proteins from larvae and adults of the porcine whipworm, *T*. *suis*, closely related to the human *T*. *trichiura*, were investigated by Leroux et al. [21], who identified a subset of proteins that promote specific anti-inflammatory functions and immunomodulatory properties. Here we present the combination of proteomic techniques, such as one-dimensional gel electrophoresis and tandem mass spectrometry as a comprehensive approach to identify *T*. *trichiura* proteins of immunodiagnostic value.

#### Vitellogenin N and VWD and DUF1943 domain containing protein

Our findings of VgNVD being a major protein in the EE proteome (Table 1) and having immunogenic value in our naturally infected monkeys (Table 3) is significant as Shears and collaborators [26] identified VgNVD in extracellular vesicles (EVs) of *T*. *muris* as a potential immunogenic candidate. Antigenic homologs of VgNVD have been identified in free-living nematodes such as *C*. *elegans*, and adult parasites secretomes of *Ascaris suum*, *Nippostrongylus brasiliensis*, *Heligmosomoides polygyrus* and *Litomosoides sigmodontis* [58–61] and also in *H*. *polygyrus* eggs [62] which confirms the significance of our results in the context of current efforts to identify potential diagnostic, vaccine or drug targets. The VgNVD was not a distinct immune complex of interest identified in the FE alone when compared to other nematode banding patterns, therefore its presence in the FE was not identified.

#### Heat shock protein 70

HSP70 and heat shock proteins, in general, have caught the attention of researchers for acting typically as immunodominant antigens eliciting strong humoral responses as major targets of host immune responses, suggesting them out as possible candidates for antiparasitic, allergic and autoimmune diseases treatments [63,64]. Our findings that HSP70 is present in the EE in low abundance is in contrast to other work where the HSP70 is amongst the most highly abundant protein identified in egg secretions of *S*. *mansoni* and *H*. *polygyrus* [17,62]. HSP70 is also heavily represented in *E*. *caproni*, *F*. *hepatica*, *H*. *polygyrus*, *Schistosoma bovis*, *T*. *trichiura*, *T*. *britovi*, and *Zygocotyle lunata* adult worms extract [14,35,43,46,65,66] which highlights that for the EE this can be a less abundant target. However, the contrasting finding of low prevalence may be due to the NE stage of the *Trichuris* eggs used in the study. Further studies are warranted to stablish this comparison. Others have reported on their immunogenicity linked to stimulation of IgG and IgM responses [41,67,68], and they have been suggested as possible vaccine targets [69].

#### Poly-cysteine and histidine tailed protein isoform 2

PCHTP-2 was the second most abundant in the EE and also present in the FE. This protein was identified as a strong immunogen of *Trichinella pseudospiralis* adult secretome [70]. Another protein of the same family, poly-cysteine and histidine-tailed metalloprotein, implicated in metal storage and/or transport, was the first member of the nematode poly-cysteine protein family described in *T*. *spiralis*. Since these proteins are unique for parasites of the Superfamily Trichinelloidea, their potential applications in diagnostics and treatment could be exploited in the future [71] and we show here that in the case of *T*. *trichiura* PCHTP-2 has a strong presence. Recent work by Bancroft [27] hypothesized that the unique structural features of the homolog protein allows binding to IL-13, which is considered the key effector cytokine responsible for *T*. *muris* expulsion, able to inhibit IL-13 function both *in vitro* and *in vivo*. Our finding that PCHTP-2 is equally abundant in EE and FE as well as a strong immunogen in our naturally infected AGMs is significant as we can confirm that this protein has a strong presence in both life cycle stages and in accordance with Bancroft [27] in the *T*. *muris* model. Our results are in agreement with presenting PCHTP-2 as a *Trichuris*-derived immunomodulatory molecule that could serve as a key target for the development of immunodiagnostics, vaccination or drug-based therapeutics.

#### Enolase and glyceraldehyde-3-phosphate dehydrogenase

We identified certain glycolytic enzymes such as enolase and GAPDH, as immunoactive components of the *T*. *trichiura* EE and FE. Both of them are present on the surface of helminths interacting with the host surface as is the case of the delicate interaction between *T*. *trichiura* and the enteric cells of the cecum. Furthermore, enolase plays a vital role in the degradation of the intracellular matrix through the activation of plasminogen facilitating the invasion, migration, and fixation in the host [15,17,33,44] all essential mechanisms to ensure *T*. *trichiura* prolonged survival. In *T*. *spiralis* [41] and *T*. *britovi* [35], this enzyme has been confirmed as immunodominant, suggesting that it may assist in tissue migration of the larvae a critical task that *T*. *trichiura* must accomplish shortly after the hatching from the egg. Enolase and heat shock proteins have also been classified as exosome markers [26,72] in accordance with our findings of enolase lacking signal peptide. Likewise, GAPDH has been previously linked to fibronectin, laminin, entactin, and collagen binding [73] and Cass and collaborators [17] suggested that in the case of *S*. *mansoni* this protein could be involved in the attachment of the eggs to host tissues or aid the passage of live eggs across host tissues to the external environment. Our results suggest GAPDH as having a relative abundance in both EE and FE that could align with Cass [17] findings and warrant further study.

The present study seeks to identify and characterize the soluble protein extracts of *T*. *trichiura* NE eggs by proteomic and immunoproteomic approaches. The *T*. *trichiura* life cycle inside the host starts with the egg hatching and the release of the larva. This period of time remains as an undiagnosed stage, while the proteins described here are directly exposed to the immune system, and as we demonstrate herein, can elicit anti-*Trichuris* antibodies by the host.

Our study is the first effort to identify the proteome of the NE *T*. *trichiura* eggs as a novel source of potential targets and provides details which might serve for improved diagnostics and immunomodulators and facilitate treatment and control of this neglected disease.

Eggs, as the infective developmental stage of the nematode, signal the host interface with their shell surface antigens and the subsequent release of larvae and associated fluids are the first stimuli to the host’s immune system. Later in infections, the NE eggs released by the females into the cecum and their secretomes would also be expected to stimulate the hosts’ immune system. The NE egg proteome we studied revealed common families of proteins which are known to play roles in energy and metabolism; the cytoskeleton, muscle and motility; proteolysis; signaling; the stress response and detoxification; transcription and translation; and lipid binding and transport. Further studies using embryonated eggs are underway in our laboratory in order to compare antigenic profiles of NE and embryonated *Trichuris* eggs and to continue identifying relevant antigenic and structural proteins. This initial list of NE *T*. *trichiura* egg proteins (proteome and antigenic profile) can be used in future research into the immunobiology and pathogenesis of human trichuriasis and the treatment of human intestinal immune-related diseases.

## Supporting information

S1 Table Proteins identified in *T*. *trichiura* non-embryonated egg extract (EE) proteome by LC-MS/MS using ProteinPilot v 4.5. and Gene Ontology database. (XLSX)

S1 Alternative Language Abstract Translation of the Abstract into Spanish by M. Trelis. (DOCX)
