# Supplementary material for: Trichuris trichiura egg extract proteome reveals potential diagnostic targets and immunomodulators
Source: PLoS Negl Trop Dis. 2021 Mar 24;15(3):e0009221. doi: 10.1371/journal.pntd.0009221 (PMC8021180; doi:10.1371/journal.pntd.0009221)
Supplement: S1 Alternative Language Abstract — (DOCX) [file pntd.0009221.s002.docx]

**Abstract (Spanish) - Translation by M. Trelis**

Los huevos embrionados de *Trichuris trichiura* son la forma infectante de este nematodo, y representan el primer estímulo para el sistema inmunitario del hospedador. El invasor intestinal, *T. trichiura*, afecta aproximadamente a 465 millones de personas alrededor del mundo, sumando 640.000 años de vida ajustados por discapacidad (AVAD o DALY, Disability Adjusted Life Years). En América Latina y el Caribe, la tricuriosis es la geohelmintiasis más prevelente (12.3%; 95% IC). Los efectos adversos de esta enfermedad, impiden el desarrollo normal de la niñez, limitando el rendimiento y la asistencia escolar, resultando en una limitada capacidad para mantener un salario mínimo en el futuro. La acumulación de estos efectos a largo plazo se traduce en un círculo de secuelas promotoras de pobreza y en un ciclo de emprobrecimiento.

Cada huevo de *T. trichiura* contiene los antígenos necesarios para enfrentarse al sistema inmunitario con una gran variedad de proteínas presentes en su cubierta, en la superficie de la larva que contiene, y en el líquido de excreción/secreción que acompaña a los componentes del huevo.

Se realizó un análisis proteómico mediante espectrometría de masas en tándem de las proteínas solubles del extracto del huevo no embrionado de *T. trichiura* aislados de monos verdes africanos (*Chlorocebus sabaeus*) infectados naturalmente con este nematodo. Se identificaron un total de 231 proteínas, 168 de ellas con funciones celulares conocidas. El proteoma reveló familias de proteínas comunes, que son conocidas por su relación con la obtención de energía, en el metabolismo; el citoesqueleto, la contracción y la movilidad; la proteólisis; la señalización; la respuesta al estrés y la detoxificación; la transcripción y la traducción; y la unión de lípidos y el transporte celular.

Junto con el estudio del proteoma del huevo no embrionado de *T. trichiura*, se investigó el perfil antigénico de las proteínas solubles, tanto del huevo como de la hembra de *T. trichiura,* sobre los anticuerpos presentes en el suero de *C. sabaeus* con tricuriosis natural. Para ello, se utilizó un enfoque inmunoproteómico con Western blot y espectrometría de masas en tándem de los geles SDS-PAGE correspondientes. La proteína vitellogenina N con dominios de VWD y DUF1943, la proteína poli-cisteína e histidina isoforma 2, la proteína de choque térmico 70, la gliceraldehido-3-fosfato deshidrogenasa, la actina y la enolasa, son las proteínas propuestas en nuestro estudio como las potencialmente inmunoactivas. Hasta la fecha, este es el primer estudio del proteoma de los huevos de *T. trichiura,* y el uso de estos como una fuente alternativa de información sobre posibles moléculas diana para inmunodiagnóstico e inmunomodulación de esta enfermedad tropical desatendida.
